# Dysregulation of NK and CD8^+^T Cells by the Microbiota Promotes the Progression of Lung Cancer

**DOI:** 10.1155/2022/7057089

**Published:** 2022-08-05

**Authors:** Shouxin Hu, Yanfang Zha, Wenwen Yang, Kele Cui, Min Cheng

**Affiliations:** ^1^Department of Geriatrics, Gerontology Institute of Anhui Province, The First Affiliated Hospital of USTC, Division of Life Sciences and Medicine, University of Science and Technology of China, Hefei, Anhui 230001, China; ^2^Anhui Provincial Key Laboratory of Tumor Immunotherapy and Nutrition Therapy, Hefei 230001, China; ^3^Cancer Immunotherapy Center, The First Affiliated Hospital of USTC, Division of Life Sciences and Medicine, University of Science and Technology of China, Hefei 230001, China; ^4^Department of Clinical Laboratory, The First Affiliated Hospital of USTC, Division of Life Sciences and Medicine, University of Science and Technology of China, Hefei 230001, China

## Abstract

The commensal microbiota is involved in maintaining local pulmonary immune homeostasis under physiological conditions. Alterations in the amount and dominant species of the microbiota can reshape the immune response of the body and lead to a variety of lung diseases, including cancer. The precise mechanisms by which microbiota regulate immune cells during the progression of lung cancer remain obscure. In this study, using a *Kras-*mutated-driven spontaneous lung cancer mouse model, we found that the depletion of microbiota can alleviate lung lesions in *Kras*-mutated mice at different stages of tumour development. Long-term antibiotic treatment significantly reduced the number NK cells and IFN-*γ* secretion and CD8^+^T cells in the lungs of wild-type (WT) mice, suggesting that the microbiota plays an important role in maintaining homeostasis of NK cells and CD8^+^T cells under normal conditions. However, in *Kras-*mutated mice, the altered pulmonary immune microenvironment resulted in a microbiota disorder and in the loss of the ability to regulate the immune responses of NK cells and CD8^+^T cells, thus promoting the occurrence and development of lung cancer. Further mechanistic studies have shown that the CXCL9-CXCR3 axis participated in the local recruitment of NK cells and CD8^+^T cells by the microbiota into lung tissues in *Kras*-mutated mice. Our findings reveal the role of the microbiota in reshaping tumour-related immune responses involving NK cells and CD8^+^T cells and shed light on the clinical immunotherapy of lung cancer.

## 1. Introduction

Lung cancer is a cancer type with the highest morbidity and mortality worldwide, with lung adenocarcinoma (LUAD) being the most common form of the disease. One of the most common genetic mutations leading to LUAD is the *Kras* mutation, whose targeted therapy is still a challenge [1, 2]. Although immunotherapies have shown sustained clinical responses in the treatment of LUAD, efficacy varies and is closely related to the quantity and properties of tumour infiltrating lymphocytes [3]. Therefore, focusing on the immune microenvironment of the lung and exploring potential immunotherapy targets is of great value in the treatment of *Kras*-driven lung cancer.

Commensal microbiota colonise various mucosal tissues of the body, including the gastrointestinal tract, oral cavity, respiratory tract, urogenital tract, and skin, providing immunomodulatory signals during the development, differentiation, and activation of immune cells, which are important for the maintenance of immune homeostasis in mucosal tissues, such as the lung [4–7]. Under healthy conditions, lipopolysaccharides and other components on the surface of the pulmonary microbiota can initiate innate adaptive immune responses in the lung that prevent the pathogen from colonizing the lung and causing lung disease [8–11]. However, the abnormal composition and quantity of the microbiota community, usually caused by long-term antibiotic use, smoking, and persistent infections, can result in an imbalanced immune response and leads to an increased susceptibility of an individual to various diseases, such as chronic obstructive lung disease, cystic fibrosis, asthma, and even cancer in severe cases [12–16]. Interestingly, in different lung cancer mouse models, the microbiota can play opposite roles in cancer progression, possibly due to its function to orchestrate the balance between tumour-promoting inflammation and antitumour immunity, depending on the specific context of the tumour microenvironment [4, 17, 18]. Although previous study has reported that the microbiota can promote the progression of lung cancer induced by the *Kras* mutation and loss of *p53* expression [17], the precise mechanism of its role in shaping the immune surveillance of tumour cells in *Kras*-driven lung cancer remains to be further explored.

The microbiota can promote the development and functional formation of lung immune cells, including natural killer (NK) cells, CD8^+^T cells, *γδ*T cells, dendritic cells (DCs), macrophages, and regulatory T cells (Treg), through complex crosstalk [6, 7, 19–21]. NK cells and CD8^+^T cells are the most important innate and adaptive immune cells in the human immune system, respectively. They can directly kill abnormal cells in the body and exert essential roles in preventing the infection and reinfection of intracellular pathogens such as viruses and bacteria and killing tumour cells. In lungs of vancomycin/neomycin-aerosolized mice, a decrease in bacterial load was associated with reduced regulatory T cells and enhanced T cell and NK cell activation that paralleled a significant reduction of melanoma B16 lung metastases [18]. However, it is not clear whether the microbiota is involved in the development of *Kras*-driven lung cancer by remodelling the functional characteristics of NK cells and CD8^+^T cells.

In this study, we investigated the complex crosstalk between the commensal microbiota and the host immune system in the development of lung cancer using a *Kras*-driven mouse model. We found that antibiotic treatment can alleviate lung lesions in *Kras* mice at different stages of cancer development. Changes in the immune microenvironment led to the disorder of the local microbiota, which cannot limit the amount and function of NK cells and CD8^+^T cells in the lung, thus promoting cancer progression. These findings revealed the role of the microbiota in reshaping tumour-related immune responses and provide new information for the immunotherapy of lung cancer.

## 2. Materials and Methods

### 2.1. Mice

*Kras^G12D^* (*Kras*) mice were purchased from the Nanjing University Model Animal Research Center (Nanjing, China), and WT mice (without *Kras* mutation) were littermates of *Kras-*mutated mice. All mice had a C57BL/6 background and were maintained under SPF and controlled conditions (22°C, 55% humidity, and 12-hour day/night cycles), and only female mice were used in all mouse experiments.

### 2.2. *In Vivo* Procedures

In spontaneous models, sex-matched WT and *Kras* mice aged 5 weeks were infected intranasally (i.n.) with HBLV-Cre-3xflag-ZsGreen-PURO Lenti-Cre (Hanbio, Shanghai, China) for induction of lung cancer as previously described [22]. The mice were fed with normal water or combined antibiotics, including 1 g/L ampicillin sodium (Sangon Biotech, Shanghai, China), 0.5 g/L vancomycin (Yuanye Bio-Technology, Shanghai, China), 1 g/L neomycin trisulfate salt hydrate (Sangon Biotech, Shanghai, China), and 1 g/L metronidazole (Sangon Biotech, Shanghai, China) in their drinking water 5 weeks before Lenti-Cre delivery, which was continued throughout the whole experiment. All experimental procedures were approved by the Ethics Committee of the University of Science and Technology of China (Hefei, China).

### 2.3. Isolation of Lung Mononuclear Cells

As previously described [23], mononuclear cells (MNCs) were isolated from the lungs by density gradient centrifugation using 40% and 70% Percoll solution (GE Healthcare, Uppsala, Sweden).

### 2.4. Flow Cytometry Analysis and Antibody Staining

The following fluorescence-labeled monoclonal antibodies were used: PE-CY7-anti-NK1.1 (PK136), FITC-anti-CD8 (53-6.7), PE-anti-CD4 (RM4-5), APC-anti-CD4 (RM4-5), and APC-anti-IFN-*γ* (XMG1.2) were purchased from BD Biosciences (Franklin Lakes, NJ, USA); FITC-anti-CD3(145-2C11), BV421-anti-TNF-*α* (MP6-XT22), and BV510-anti-CD8 (53-6.7) were purchased from BioLegend (San Diego, USA). For the cell phenotype assays, 1 × 10^6^ cells were blocked with 10 *μ*L rat serum for 30 min at 4°C and then stained with the indicated antibodies for 30 min at 4°C in the dark. For the intracellular cytokine assays, cells were stimulated with the Cell Activation Cocktail (including 30 ng/mL PMA and 1 *μ*g/mL ionomycin) (BioLegend, San Diego, USA) and 10 *μ*g/mL monensin (Sigma, St Louis, USA) for 4 h and were labelled with surface markers. Then cells were fixed, permeabilized, and labelled with the indicated intracellular antibodies for 30 min at 4°C in the dark. All data were acquired using an Aria II FACS flow cytometer (Becton Dickinson, Franklin Lakes, USA) and analysed using FlowJo 10.0 software (Treestar, Ashland, USA).

### 2.5. Quantitative Real-Time Polymerase Chain Reaction

Total RNA was extracted from the lung tissue using the TransZol Up Plus RNA Kit (TransGen Biotech, Beijing, China) and reverse-transcribed as previously described [4]. The gene expression levels were measured by q-PCR using the commercially available SYBR Green Premix (Accurate Biology, Changsha, China) according to the manufacturer's instructions. Gene expression levels were quantified using the *ΔΔ*Ct method. The following primers were synthesized by Tsingke Biology (Nanjing, China): *β*-actin (forward 5′-CCACTGTCGAGTCGCGTCC-3′, reverse 5′-ATTCCCACCATCACACCCTGG-3′), CXCL9 (forward 5′-AGTCCGGATCTAGGCAGGTT-3′, reverse 5′-GAGGCACGATCCACTACAAA-3′), CXCL10 (forward 5′-CCTATGGCCCTCATTCTCAC′, reverse 5′-CGTCATTTTCTGCCTCATCC-3′), and CXCR3 (forward 5′-GGCTCCTCCTGACAACAGAC-3′, reverse 5′-TGCCCAGGCTGACTTCATAC-3′).

### 2.6. Histological Examination

For histological examination, mouse left lung samples were fixed in 10% neutral buffer formalin and embedded in paraffin. Sections 4 *μ*m thick were stained with hematoxylin and eosin. Sections were photographed using an Olympus IX73 microscope (Olympus, Tokyo, Japan).

### 2.7. Statistical Analysis

Student's *t*-test was performed to analyse the significance of differences between two groups, and a value of *p* < 0.05 was considered statistically significant. All data were analysed using GraphPad Prism 6.0 software (GraphPad Software, Inc., San Diego, CA) and are expressed as means ± SEM.

## 3. Results

### 3.1. The Microbiota Promoted Tumour Progression in *Kras*-Driven Lung Cancer

**Figure 1:**
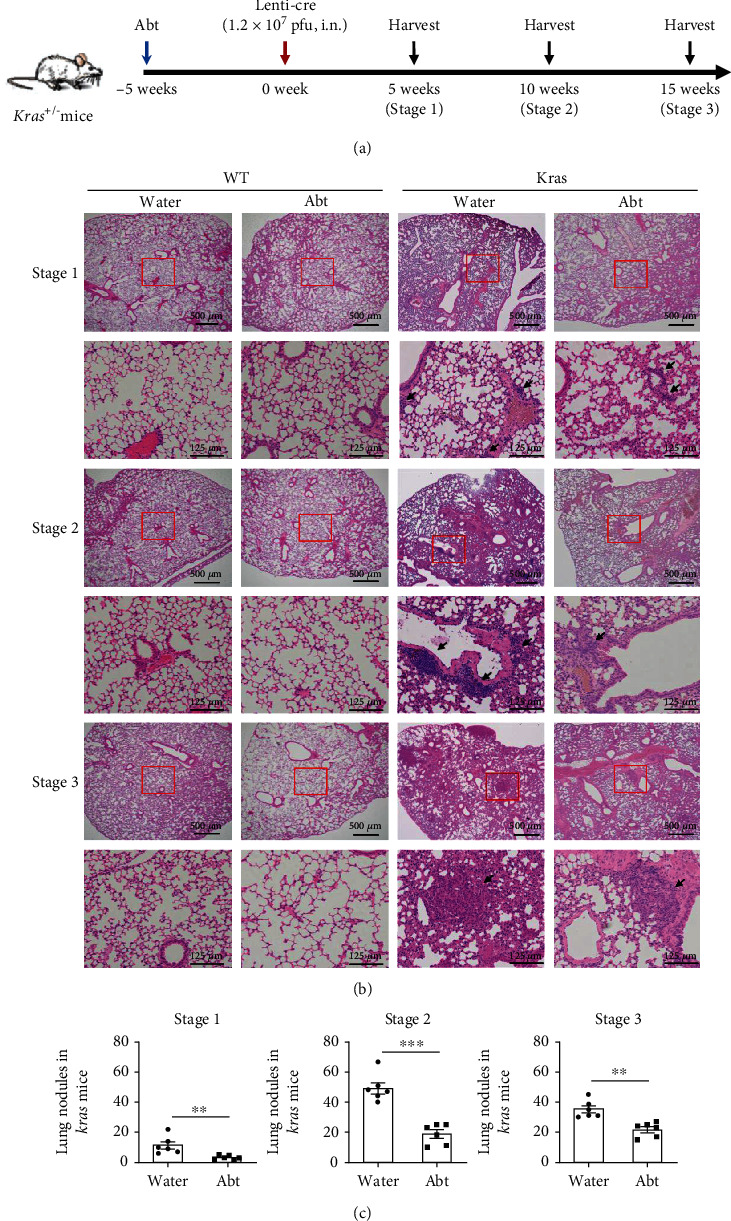
Progression of lung cancer was significantly inhibited in Abt-*Kras* mice. (a) *Kras*-mutated mice and paired control wild-type (WT) mice were infected intranasally (i.n.) with Lenti-Cre after being treated with combined antibiotics (Abt) in their drinking water for 5 weeks. The mice continued to receive Abt water throughout the experiments and tissues were harvested at 5 (stage 1), 10 (stage 2), and 15 (stage 3) weeks, respectively, after activation of the *KRAS* gene. Another two groups of *Kras*-mutated mice and WT mice were fed normal drinking water as controls. (b) Representative histology of the H&E-stained lungs of *Kras* mice and WT mice that receive Abt or normal drinking water at different stages of lung cancer. The number of tumour nodules in the two groups of Kras mice indicated in (b) is shown in (c). Each symbol in (c) represents an individual mouse; *n* = 6. Data are presented as mean ± SEM. ^∗∗^*p* < 0.01 and ^∗∗∗^*p* < 0.001.

To investigate the effects of commensal microbiota on the occurrence and development of primary lung cancer, we used a Cre-inducible *Kras*^G12D^ (*Kras*) knock-in lung cancer mouse model [1]. *Kras* and WT mice were treated with antibiotics to deplete their microbiota 5 weeks before Lenti-Cre delivery and continued to receive antibiotics diluted in water throughout the subsequent experiments; tumour development was evaluated using a three-stage system as previously reported [1] (Figure 1(a)). In addition, another two groups of *Kras-*mutated and WT mice were fed normal drinking water as controls. At 5 weeks after Lenti-Cre delivery (stage 1), atypical adenomatous hyperplasia or small adenomas were observed as the earliest lesions in *Kras*-mutated mice models. Conversely, the antibiotic treated-*Kras* (Abt-*Kras*) group showed fewer tumour nodules. At 10 weeks after Lenti-Cre delivery (stage 2), transformed lung epithelial cells in *Kras* mice underwent benign proliferation and larger adenomas and uniform nuclei were evident. Compared to the *Kras-*mutated mice, the adenomas in the Abt-*Kras* group were fewer and smaller. At 15 weeks after Lenti-Cre delivery (stage 3), which was a malignant phase, typical pleomorphic nuclei adenocarcinomas appeared in *Kras-*mutated mice. As the pulmonary nodules fused with each other, *Kras-*mutated mice showed larger but fewer lung tumour nodules compared to stage 2 tumours. Similarly, the lungs of Abt-*Kras* mice at stage 3 presented smaller lesions (Figures 1(b) and 1(c)). These results indicated that the microbiota plays an essential role in promoting the development of lung cancer in mice.

### 3.2. The Microbiota Lost the Ability to Regulate NK Cells and CD8^+^T Cells during the Development of Lung Cancer

**Figure 2:**
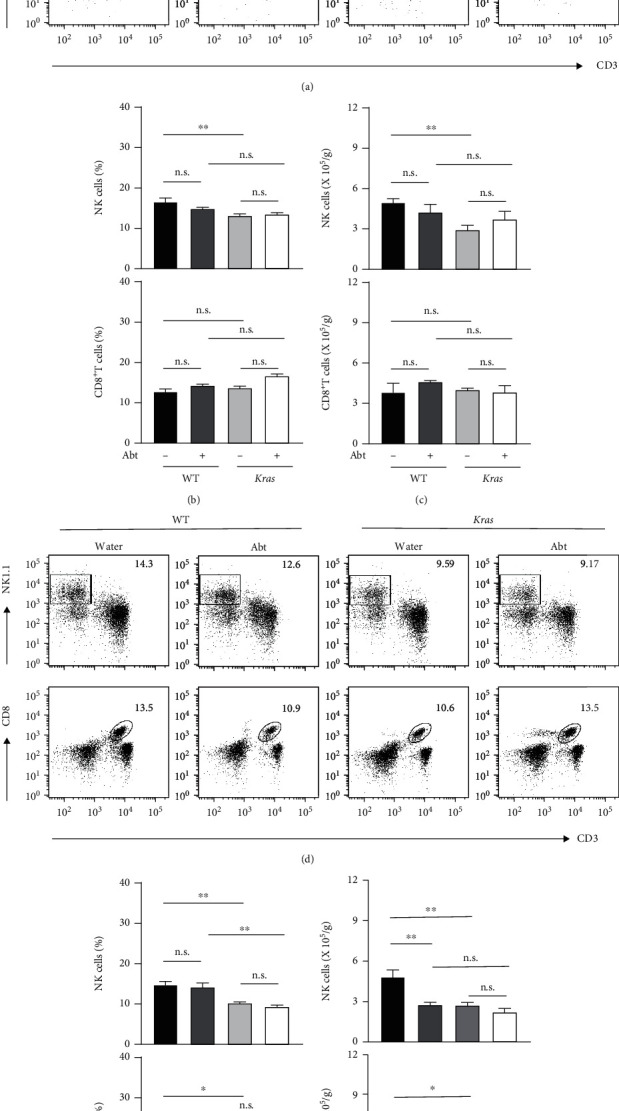
The effects of the commensal microbiota on the percentage and number of NK cells and CD8^+^T cells in different stages of lung cancer. (a–c) Representative FCM plots (a) and quantification of the percentages (b) and numbers (c) of lung CD45^+^CD3^−^NK1.1^+^ NK cells and CD45^+^CD3^+^CD8^+^ cells at stage 1 of cancer. (d–f) Representative dot plots (d) and quantifications of the percentages (e) and numbers (f) of lung NK and CD8^+^T cells at stage 3 cancer, *n* = 5 − 6. Data are presented as mean ± SEM. ^∗^*p* < 0.05 and ^∗∗^*p* < 0.01. n.s.: not significant.

NK cells and CD8^+^T cells are the main executors in the killing of abnormal cells and tumour cells and play an important role in the anti-infection and antitumour process. To explore whether the microbiota affect the occurrence and development of lung cancer by regulating NK and CD8^+^T cell levels, lung mononuclear cells were isolated and analysed by flow cytometry at tumour stages 1 and 3, respectively. Antibiotic treatment showed no significant effect on the amount of NK cells and CD8^+^T cells in *Kras-*mutated or WT mice at stage 1. Compared to the WT mice, the percentage and number of NK cells, but not CD8^+^T cells, in *Kras*-mutated mice decreased significantly at this stage (Figures 2(a)–2(c)). However, as the tumour progressed, the percentage and number of NK cells and CD8^+^T cells in *Kras-*mutated mice had an obvious decrease compared to WT mice in stage 3 tumours (Figures 2(d)–2(f)). Although long-term antibiotic treatment significantly reduced the number of lung NK cells and CD8^+^T cells in WT mice, it had no obvious effect on the levels in *Kras-*mutated mice (Figures 2(d)–2(f)).

We also detected variations in pulmonary CD4^+^T cells in the four groups of mice at stages 1 and 3. The results indicated that there were no significant differences in the percentage or amount of CD4^+^T cells in the lungs of Abt-WT and Abt-*Kras* mice compared to the control groups at stage 1 (Figure S1a). At stage 3, although antibiotic treatment did not affect the percentage of CD4^+^ T cells in the lung, it significantly reduced their number in the lungs of WT and *Kras-*mutated mice (Figure S1b). Meanwhile, we found that compared to WT mice, the amount of pulmonary CD4^+^T cells in *Kras-*mutated mice was not influenced during of lung cancer progression (Figure S1a and b). These results suggested that the microbiota exerts vital roles in maintaining the number of immune cells in the lungs of mice, including NK, CD8^+^T, and CD4^+^T cells. However, in the progression of lung cancer in *Kras* mice, dysregulation of pulmonary immune homeostasis results in the inability of the microbiota to maintain the number of NK cells and CD8^+^T cells, rather than CD4^+^T cells, in the local microenvironment with powerful antitumour functions, promoting disease progression.

**Figure 3:**
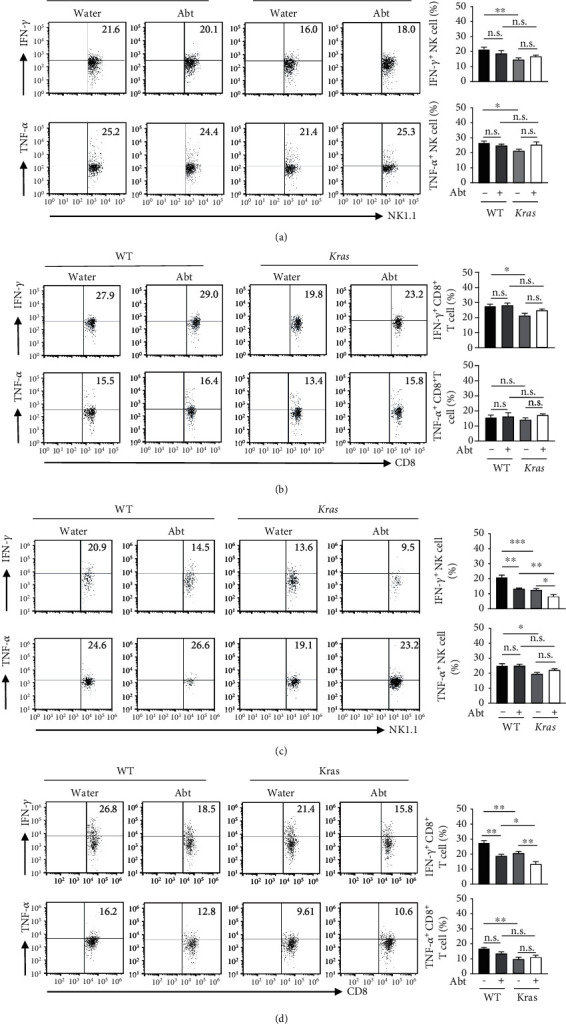
Production of IFN-*γ* was impaired in NK cells and CD8^+^T cells at stage 3 of lung cancer in Abt-*Kras* mice. (a and b) Representative dot plots and quantifications of IFN-*γ* and TNF-*α* expression in lung NK cells (a) and CD8^+^T cells (b) at stage 1 cancer. (c and d) Representative dot plots and quantifications of IFN-*γ* and TNF-*α* expression in lung NK cells (c) and CD8^+^T cells (d) at stage 3 cancer, *n* = 4 − 6. Data are presented as mean ± SEM. ^∗^*p* < 0.05, ^∗∗^*p* < 0.01, and ^∗∗∗^*p* < 0.001. n.s.: not significant.

NK cells and CD8^+^T cells participate in tumour progression by secreting cytokines such as interferon- (IFN-) *γ* and tumour necrosis factor- (TNF-) *α*. To explore whether the microbiota affects the secretion of cytokines from pulmonary NK cells and CD8^+^T cells, we also measured IFN-*γ* and TNF-*α* secretion in the four groups of mice at stages 1 and 3. The results showed that in stage 1, the IFN-*γ* and TNF- *α* production of lung NK cells in *Kras*-mutated mice was significantly reduced compared with WT mice. Antibiotic treatment only reduced IFN-*γ*, but not TNF-*α*, secretion in pulmonary NK cells from WT mice, while it had no obvious effect on the levels of these two cytokines in NK cells from *Kras*-mutated mice (Figure 3(a)). The IFN-*γ*, rather than TNF-*α*, secretion of lung CD8^+^T cells in *Kras-*mutated mice in early tumourigenesis was significantly reduced compared to WT mice. Nevertheless, antibiotic treatment did not affect the production of the two cytokines in lung CD8^+^T cells of WT and *Kras-*mutated mice (Figure 3(b)). At stage 3, tumour progression further downregulated IFN-*γ* levels of NK cells in *Kras-*mutated mice, and antibiotic treatment accelerated the IFN-*γ* decrease, while there was no significant change in TNF-*α* secretion of NK cells in the four groups (Figure 3(c)). In addition, we found that tumour progression significantly downregulated the IFN-*γ* and TNF-*α* secretion levels of CD8^+^T cells in *Kras-*mutated mice at this stage, while long-term antibiotic treatment only inhibited their secretion of IFN-*γ*, but not TNF-*α* (Figure 3(d)).

### 3.3. The CXCL9-CXCR3 Axis Was Involved in the Local Recruitment of NK Cells and CD8^+^T Cells by the Microbiota into Lung Tissues during Tumour Progression

**Figure 4:**
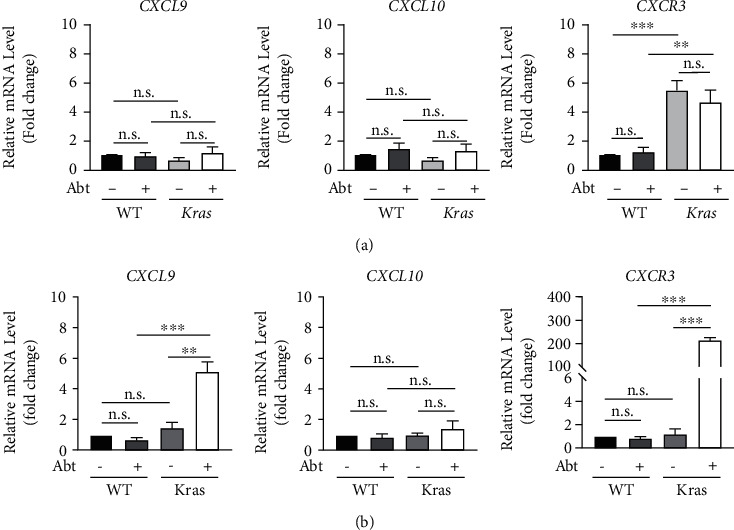
High expression of CXCL9 and CXCR3 in the lung of Abt-*Kras* mice. CXCL9, CXCL10, and CXCR3 mRNA levels in the lungs at stage 1 (a) and stage 3 (b) cancer were measured by real-time PCR, *n* = 4–5. Data are presented as mean ± SEM. ^∗∗^*p* < 0.01 and ^∗∗∗^*p* < 0.001. n.s.: not significant.

Chemokines and their receptors in the tissue microenvironment play an important role in regulating the migration of immune cells to local regions, primarily regulating the migration, differentiation, and activation. Previous study has indicated that immune reactivity occurs through the CXCL9/10-CXCR3 axis by recruiting immune cells, such as cytotoxic lymphocytes (CTLs), NK cells, NKT cells, and macrophages [24]. To investigate whether the microbiota influenced the local migration of NK cells and CD8^+^T cells to lung tissues by influencing the expression of the CXCL9/10-CXCR3 axis during the progression of lung cancer in mice, we evaluated CXCL9, CXCL10, and CXCR3 mRNA levels in lung tissues of Abt-WT and *Kras-*mutated mice and paired controls at stage 1 and 3 tumours. The results showed that CXCR3 mRNA levels were obviously increased in the lungs of *Kras-*mutated mice at the early stage of tumourigenesis regardless of microbiota deletion compared to the WT group, while the expression of pulmonary CXCL9 and CXCL10 mRNA did not differ between the four groups of mice (Figure 4(a)). At stage 3, long-term antibiotic treatment significantly increased CXCL9 and CXCR3 mRNA levels in the lungs of *Kras-*mutated mice but had no effect on WT mice. Meanwhile, the absence of microbiota did not affect the expression of CXCL10 mRNA in the lungs of WT and *Kras-*mutated mice (Figure 4(b)). These results suggested that the lung microenvironment can promote the local invasion of immune cells into the tumour by upregulating the expression of CXCR3 in the early stage of tumour development, thus playing an antitumour function. However, with tumour progression, changes in the pulmonary immune microenvironment led to dysregulation of symbiotic bacteria, which prevented immune cell migration to lung tissues by inhibiting the CXCL9-CXCR3 axis, promoting tumour occurrence and development.

## 4. Discussion

As a mucosal tissue with the largest surface area in the body, the mucosal surfaces of the lung are exposed to a variety of airborne microbes and environmental insults through inhalation [17]. Direct interaction of the mucosal microbiota with developing tumour cells and the local immune system and indirect regulation of the systemic immune response by the distal intestinal microbiota are crucial factors affecting the progression of lung cancer. Tumour-associated barrier defects and airway obstruction can result in altered bacterial clearance and altered microbe growth conditions, further affecting the status and function of immune cells. Our study found that under physiological conditions, the microbiota participates in maintaining the normal pulmonary immune microenvironment of mice. However, when a tumour occurs, the microbiota in our model animals lost its ability to regulate the number and function of NK cells and CD8^+^T cells, thus promoting tumour development.

The lungs harbour a unique microbiota, which is influenced by microbial signals from intestine through the gut-lung axis [25]. Many gastrointestinal disorders have manifestations in the respiratory tract. Alterations in the quantity and composition of the gut microbiota are associated with the host immune response against certain lung diseases, such as influenza A virus infection and allergens [26, 27]. It should be noted that commensal bacteria are critical regulators in shaping antitumour immunosurveillance of distal tissues, including various primary and metastatic liver and lung tumours [17, 28]. Unlike increasing the antitumour immune response by controlling bile acid metabolism in liver cancer [28], our current study found that the microbiota significantly promoted the progression of *Kras-*driven lung cancer in mice, suggesting that changes in the gut microbiota can cause opposite effects in different organs (Figure 1).

We previously reported that commensal bacteria shaped immune surveillance efficiency in B16/F10 melanoma and Lewis lung carcinoma, while another group provided evidence that the microbiota promoted lung cancer development induced by the *Kras* mutation and the loss of *p53*; interestingly, both effects involved the regulation of the microbiota on *γδ*T17 cells [4, 17]. However, little is known about the dynamic effects of the microbiota on NK cells and CD8^+^T cells on lung cancer development. In this study, we revealed that a short period of microbiota deletion did not affect the amount of NK cells and CD8^+^T cells, while long-term treatment of combined antibiotic could obviously reduce the absolute numbers of NK cells and CD8^+^T cells in the lungs of WT mice, indicating that the microbiota plays an important role in maintaining pulmonary immune homeostasis (Figure 2). However, although the number of lung NK cells and CD8^+^T cells in *Kras-*mutated mice decreased significantly with tumour progression compared to WT mice, antibiotic treatment did not exacerbate the decrease (Figures 2(d)–2(f)). These results suggested that changes in the pulmonary microenvironment of *Kras-*mutated mice can lead to dysregulation of the microbiota, which in turn loses its ability to regulate the number and function of immune cells in local tissues. This may partly explain the significant retardation of tumour progression in *Kras-*mutated mice with loss of microbiota. The CXCL9-CXCR3 axis involves recruiting immune cells into the tumour microenvironment in a variety of solid tumours, such as epithelial ovarian cancer, non-small-cell lung cancer, melanoma, breast cancer, and colon cancer [29–35]. In the current study, we found that the expressions of CXCL9 and CXCR3 mRNA increased significantly in the lungs of Abt-*Kras* mice compared to paired controls as tumour progressed, and this may be the main reason for the constant amount of NK cells and CD8^+^T cells in their lung (Figure 4).

As a typical proinflammatory factor, IFN-*γ* has been shown to have a tumour-promoting effect by recent studies, in addition to its classical immunomodulatory, antiviral, and antitumour functions. For example, IFN-*γ* can promote tumour cell survival, accelerate tumour cell metastasis, reduce CD8^+^T cell infiltration, and mediate CD8^+^T cell apoptosis [36–39]. Furthermore, in a variety of diseases, such as type I diabetes and papilloma, IFN-*γ* synergistically promotes IL-17-related inflammatory signaling pathways and promotes disease progression [40, 41]. In particular, Jin et al. [17] proved that the microbiota can promote the secretion of *γδ*T cells, further accelerating inflammation and tumour cell proliferation in lung cancer driven by the *Kras* mutation and the loss of *p53*. Interestingly, in the current study, we observed an obvious decrease in IFN-*γ* secretion by NK cells and CD8^+^T cells in the lungs of *Kras* mice with long-term microbiota depletion, suggesting that the main function of IFN-*γ* in the lung microenvironment may be to synergize/promote IL-17A-associated inflammation, thus promoting the development of lung cancer (Figure 3). However, the exact mechanism of the roles of microbiota-regulated NK cells and CD8^+^T cells in lung cancer progression remains to be further explored, especially since the antitumour effect of IFN-*γ* cannot be ruled out.

## 5. Conclusions

Our study indicated that changes in the lung microenvironment of *Kras-*mutated mice can lead to a disorder of the microbiota in the body, resulting in the dysregulation of NK cells and CD8^+^T cells, thus promoting the appearance and development of tumours. Further mechanistic investigations showed that the CXCL9-CXCR3 axis plays a key role in the local recruitment of NK cells and CD8^+^T cells by the microbiota into lung tissues. This study sheds light on the role of microbiota in reshaping the immune response during tumour development and provides a new perspective for the clinical immunotherapy of lung cancer.

## Data Availability

All data generated or analysed in this study are available from the corresponding author on reasonable request.

## Abbreviations

Abt-*Kras*: Antibiotic treated-*Kras*
CTLs: Cytotoxic lymphocytes
DCs: Dendritic cells
IFN: Interferon
i.n.: Intranasally
LUAD: Lung adenocarcinoma
MNCs: Mononuclear cells
NK: Natural killer
Treg: Regulatory T cells
TNF: Tumour necrosis factor.

## Supplementary Materials

Supplementary Materials See Figure S1 in the Supplementary Material.
