# Supplementary material for: Dysregulation of NK and CD8+T Cells by the Microbiota Promotes the Progression of Lung Cancer
Source: J Immunol Res. 2022 Aug 5;2022:7057089. doi: 10.1155/2022/7057089 (PMC9410859; doi:10.1155/2022/7057089)
Supplement: Supplementary Materials — See Figure S1 in the Supplementary Material. [file 7057089.f1.docx]

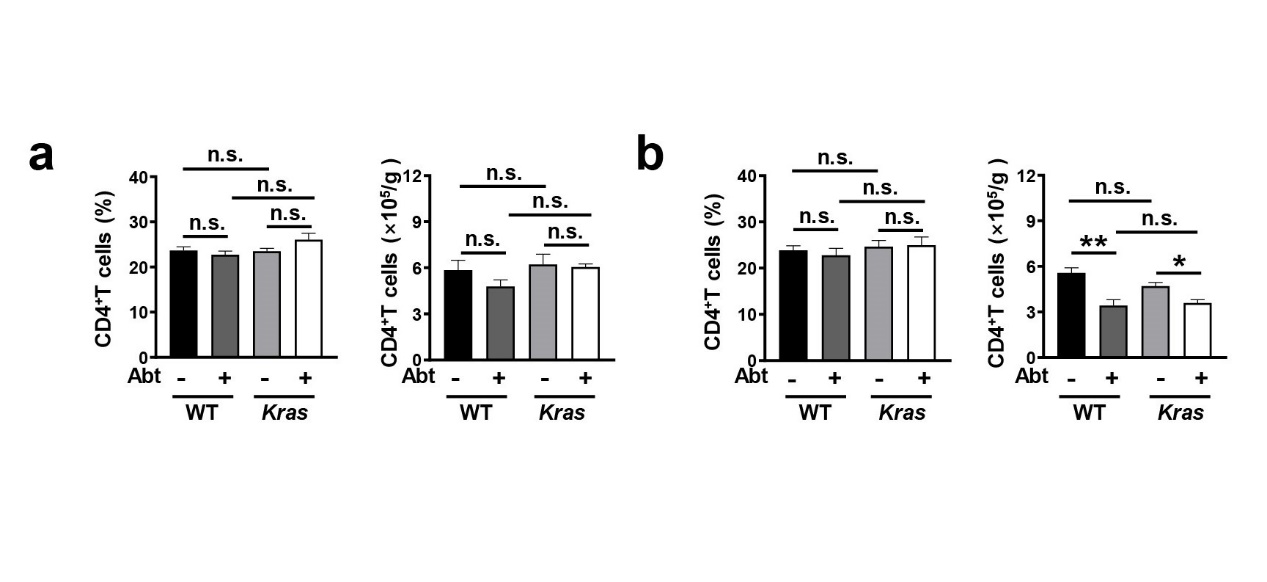


**Figure S1**. The effects of commensal microbiota on the percentage and number of CD4^+^T cells at different stages of lung cancer. Related to Figure 2. Quantification of the percentage and number of CD45^+^CD3^+^CD4^+^T cells in the lung at the stage 1 **(a)** and 3 **(b)** of lung cancer, n=4-6. Data are presented as mean ± SEM. *p<0.05, **p<0.01, and n.s., not significant.
